# A Rare Case of Acute Mesenteric Ischemia in the Setting of COVID-19 Infection

**DOI:** 10.7759/cureus.14174

**Published:** 2021-03-29

**Authors:** Neeharika Krothapalli, Jason Jacob

**Affiliations:** 1 Neurology, University of Connecticut, Farmington, USA; 2 Internal Medicine, Hartford Hospital, Hartford, USA

**Keywords:** covid-19, acute mesenteric ischemia, gastrointestinal manifestations

## Abstract

Severe respiratory syndrome coronavirus 2 (SARS-CoV-2) is well known for causing respiratory and other extrapulmonary manifestations. Patients infected with coronavirus disease 2019 (COVID-19) may demonstrate atypical presentations with gastrointestinal symptoms. Clinicians managing these patients should reserve a high index of suspicion for the rare complication of acute mesenteric ischemia (AMI). It is a challenging diagnosis that is often missed when presenting symptoms are subtle and nonspecific like nausea, emesis, or diarrhea. Outcomes are typically catastrophic and fatal as bowel ischemia progresses to necrosis but may be averted with timely diagnostic and therapeutic methods to swiftly restore blood flow.

## Introduction

Severe acute respiratory syndrome coronavirus 2 (SARS-CoV-2) and its disease, coronavirus disease 2019 (COVID-19) can manifest as a wide spectrum from asymptomatic infection and mild respiratory symptoms to multisystem organ dysfunction. The most common presenting symptoms include fever, cough, myalgia, headache, dyspnea, diarrhea, nausea, vomiting, and loss of smell or taste [1]. Severe complications implicated in COVID-19 are acute respiratory distress syndrome, arrhythmias, cardiac injury, shock, and acute stroke [2]. Although a myriad of thromboembolic complications including deep vein thrombosis and pulmonary embolism are increasingly recognized, acute mesenteric ischemia (AMI) is a rare but life-threatening entity that presents a diagnostic challenge for clinicians. It is a vascular emergency with an overall mortality of 60%-80% and necessitates a prompt diagnosis for timely intervention [3]. We present a rare case of AMI triggered by severe COVID-19 infection in an older patient.

## Case presentation

A 76-year-old female with a past medical history of coronary artery disease status post percutaneous coronary intervention (PCI), heart failure with preserved ejection fraction (HFpEF), atrial fibrillation not on anticoagulation due to recent gastrointestinal bleed, hypertension, type 2 diabetes mellitus, and prior history of abdominal reconstructive surgery 20 years ago with ostomy for two years, revision and chronic umbilical hernia presented with a one-week history of worsening shortness of breath. She became acutely dyspneic while climbing a flight of stairs, prompting her to come to the hospital. She was diagnosed with COVID-19 infection a week prior and treated with dexamethasone for 10 days. The patient denied fever, cough, chest pain, abdominal pain, nausea, vomiting, diarrhea, melena, hematochezia, or pain in response to food intake. Initial vital signs were significant for a heart rate of 180 beats per minute, respiratory rate of 30 breaths per minute, blood pressure of 211/111 mmHg, and oxygen saturation of 85% on room air. Physical examination revealed an irregular rhythm with tachycardia, decreased breath sounds, and inferiorly protruding umbilical hernia. Abdominal examination was benign with no distension, rigidity, or guarding.

Laboratory investigations were remarkable for white blood cell (WBC) count of 9 Thou/uL, lactate of 3.1 mmol/L, normal troponin (<0.03 ng/mL), prothrombin time of 12.3 seconds, international normalized ratio (INR) of 1.1, sodium of 127 mmol/L, creatinine of 1.4 mg/dL, proBNP of 10487 pg/mL and elevated acute phase reactants (D-dimer 2159 mcg/L, ferritin 468 ng/mL, C-reactive protein 7.97 mg/L, procalcitonin 0.40 ng/mL). The patient was administered 4 L of supplemental oxygen via nasal cannula, which improved her oxygen saturation to 97%. She was admitted with acute hypoxic respiratory failure secondary to COVID-19, hypertensive urgency, and hyponatremia. She was also placed on a nitroglycerin drip and transitioned to antihypertensive medications as her blood pressure stabilized. Her hyponatremia was managed with fluid restriction.

Her course was complicated by rapid atrial fibrillation requiring metoprolol and resumption of apixaban as benefits outweighed risks. While working with occupational therapy during her stay, the patient experienced sudden right facial droop, right upper extremity weakness, and new-onset aphasia with a National Institute of Health Stroke Scale (NIHSS) of 7. She demonstrated blood glucose of 170 mg/dL, blood pressure of 123/55 mmHg, and atrial fibrillation on an electrocardiogram (EKG). Transthoracic echocardiography noted an ejection fraction of 50%, mild calcific mitral stenosis, and regurgitation. Neurology was consulted and etiology was determined to be cardioembolic in the setting of being off anticoagulation as well as COVID-19 hypercoagulability. The patient was not a candidate for thrombolytic therapy as apixaban was administered within 12 hours. She underwent a cerebral angiogram and mechanical thrombectomy for left M1 occlusion.

**Figure 1:**
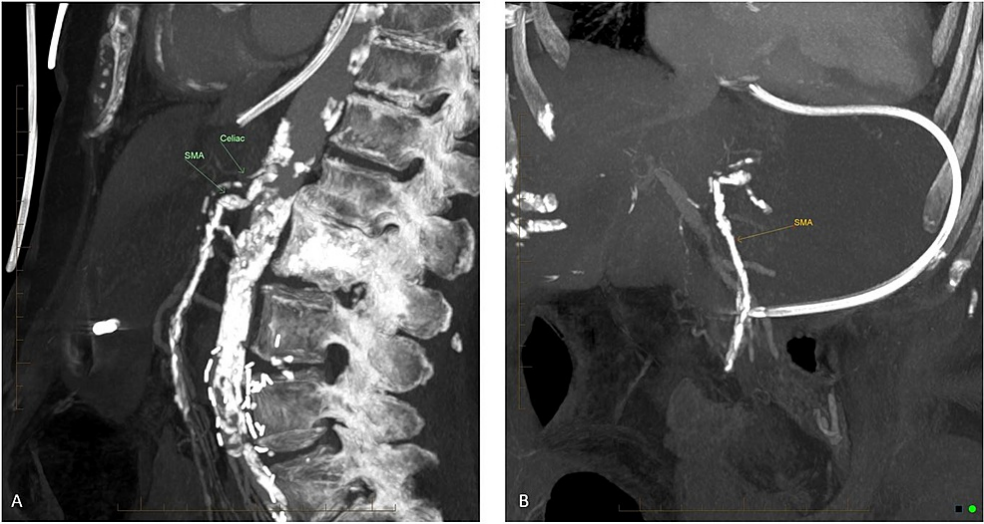
Sagittal view (A) and (B) CT abdomen and pelvis demonstrating occlusion of SMA and celiac artery with the absence of contrast. SMA, superior mesenteric artery

**Figure 2:**
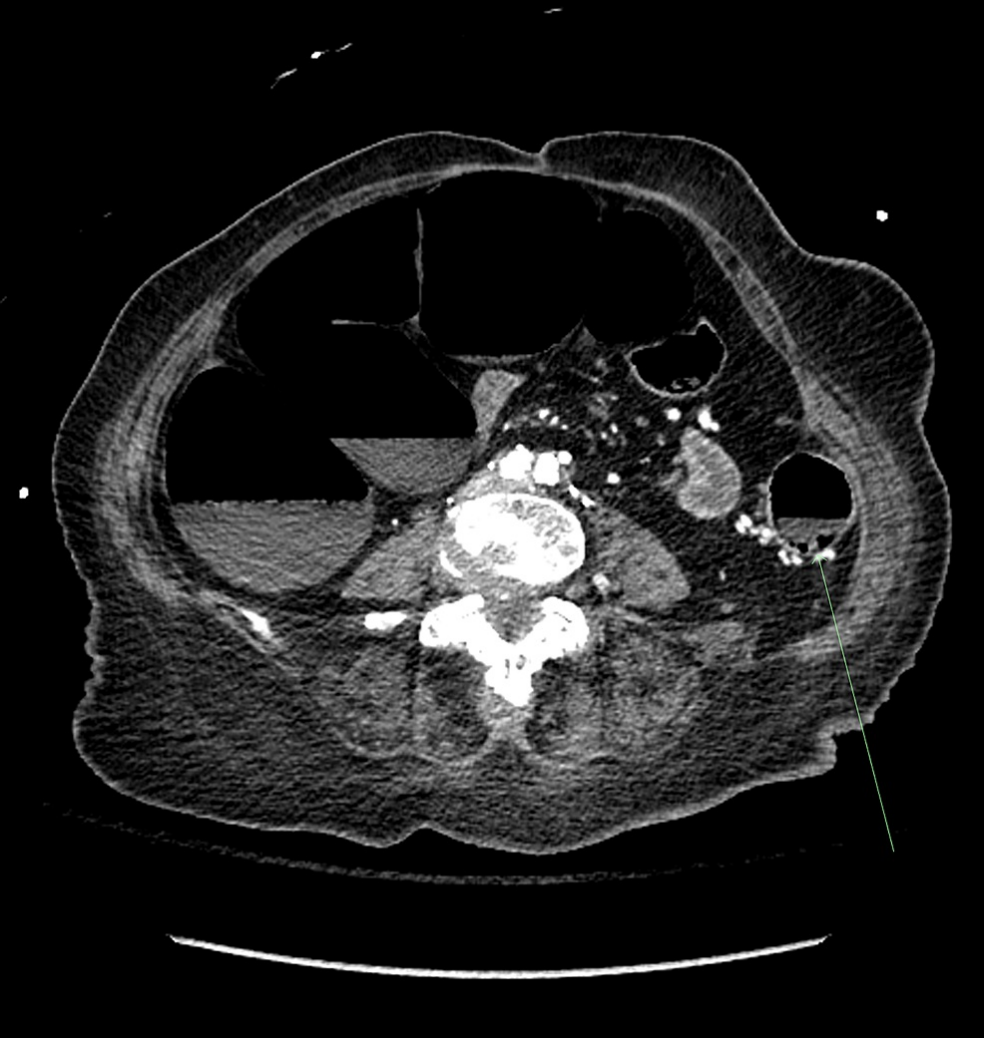
Axial view CT with dilated loops of bowel with arrow noting the area of pneumatosis.

Her hospitalization was further complicated with worsening acute hypoxic respiratory decompensation from COVID-19 pneumonia and heart failure exacerbation requiring intermittent diuresis with furosemide. She was treated with antibiotics for recurrent fevers and superimposed bronchopulmonary pneumonia. Her oxygen requirements could not be weaned down. She started to have sporadic nausea and non-bloody diarrhea that was being managed with supportive care. On day 14, the patient rapidly deteriorated as she developed an acutely distended and tender abdomen with two new bulging masses in 30 minutes lateral to her known umbilical hernia. CT of the abdomen and pelvis with contrast demonstrated celiac artery and superior mesenteric artery occlusion with intestinal ischemia (Figures 1A,B-2). She was found to have lactic acidosis at 5.9 mmol/L and required fluid resuscitation for hemodynamic support. After consultation with general and vascular surgery, the patient was determined not to be a candidate for surgical intervention given the extent of abdominal injury and poor prognosis. In concert with the goals of care discussed with her family, she was managed conservatively and transitioned to comfort care before she expired.

## Discussion

Acute mesenteric ischemia is a fatal abdominal emergency with an acute onset of symptoms and rapid clinical deterioration. A high index of suspicion with timely diagnostic and therapeutic intervention is paramount in reducing the high mortality associated with AMI. Risk factors for the development of this condition include advanced age, atrial fibrillation, cardiac valvular disease, congestive heart failure, and recent myocardial infarction which increase the potential for thrombotic occlusion or embolism [4]. The superior mesenteric artery (SMA) is especially vulnerable to emboli that can lodge because of its oblique angle from the aorta. The early signs of mesenteric ischemia are severe upper or central abdominal pain out of proportion to physical findings, nausea, vomiting, and bowel evacuation from increased intestinal activity [5]. Some patients with SMA thrombosis may report prodromal symptoms of postprandial abdominal pain, food aversion, and weight loss. Bowel ischemia can rapidly progress to infarction and irreversible necrosis, characterized by hemodynamic instability, peritoneal signs, and septic shock with multiorgan failure [6]. Bloody bowel movements are less common but may be observed with advanced mucosal infarction.

A compatible history and physical examination in the setting of predisposing risk factors is the cornerstone to early detection of this devastating condition. A definitive diagnosis of AMI is made with imaging using high-resolution CT angiography to demonstrate occlusion within the mesenteric arteries. Laboratory findings reveal metabolic acidosis with elevated anion gap and lactate levels, hemoconcentration, and leukocytosis. Hyperkalemia and hyperphosphatemia may also be identified during the late course of bowel infarction [7]. Our patient demonstrated elevated lactate and persistent leukocytosis.

Considering thrombotic complications such as stroke, acute coronary syndrome and acute limb ischemia have been reported with COVID-19 infection, an unexplained worsening clinical picture particularly in this patient population may signify the rare complication of AMI. Thereby, clinicians should have a low threshold of suspicion in critically ill patients with COVID-19 that develop gastrointestinal manifestations like severe abdominal pain. Although the underlying pathogenesis is poorly understood, it is postulated that COVID-19 causes a transient hypercoagulable state [8]. SARS-CoV-2 may target enterocytes of the small bowel and lead to the expression of angiotensin-converting enzyme (ACE) 2, resulting in endothelial cell damage and increasing procoagulant factors like von Willebrand Factor to cause vascular thrombosis [9-10].

The goal of treatment is to restore intestinal blood flow as promptly as possible after initial management with systemic anticoagulation and empiric broad-spectrum antibiotics [11]. Surgical resection of necrotic bowel, supportive measures including gastrointestinal decompression, fluid resuscitation, and hemodynamic support are the backbone of AMI therapy [12]. Survival is approximately 50% when the diagnosis is made within one day but drops to less than 30% with delayed diagnosis [13]. Perioperative mortality in patients who undergo revascularization ranges between 44% and 90% [13]. There is limited data on long-term sequelae of successful revascularization, but a small subset of patients may develop short-gut syndrome requiring small bowel transplantation or total parenteral alimentation. In patients without contraindications, pharmacological thromboprophylaxis with regular monitoring of a coagulation panel guides the prevention and treatment of thromboembolic complications of the disease [14].

A limitation in our case study may be her considerable burden of atherosclerotic disease which could have led to the occurrence of AMI. However, our patient concomitantly experienced several major thrombotic events from ischemic stroke to AMI within a very short timeframe which makes it challenging to explain with just her predisposing factors. Given the recent diagnosis of COVID-19, it may be the culprit of her increased susceptibility to these events during hospitalization. The concept of endothelitis is an emerging phenomenon that explains why COVID-19 has such a disproportionate effect on patients with existing cardiovascular comorbidities, leading to microthrombus formation and organ ischemia. Some rationalize that stabilizing the endothelium and targeting viral replication with anti-inflammatory anti-cytokine medications, ACE inhibitors, and statins may prove beneficial in vulnerable patients with pre-existing endothelial dysfunction [15], which requires further investigation.

## Conclusions

Although AMI is a rare complication of COVID-19 infection, physicians should be vigilant of this entity because of the catastrophic consequences of an undetected or late diagnosis. This case highlights the importance of having a broad differential and high suspicion for AMI when a patient with COVID-19 has an unexplained clinical picture with gastrointestinal manifestations. Adequate prophylactic measures should be implemented in eligible patients and prompt imaging should be used to help guide diagnosis. Ultimately, timely and aggressive intervention may improve the chances of survival.
